# Oxidative Stress in Cardiovascular Diseases: Still a Therapeutic Target?

**DOI:** 10.3390/nu11092090

**Published:** 2019-09-04

**Authors:** Thomas Senoner, Wolfgang Dichtl

**Affiliations:** Department of Internal Medicine III, Cardiology and Angiology, Innsbruck Medical University, Anichstraße 35, 6020 Innsbruck, Austria

**Keywords:** antioxidants, oxidative stress, nutraceuticals, cardiovascular diseases

## Abstract

Cardiovascular diseases (CVD) are complex entities with heterogenous pathophysiologic mechanisms and increased oxidative stress has been viewed as one of the potential common etiologies. A fine balance between the presence of reactive oxygen species (ROS) and antioxidants is essential for the proper normal functioning of the cell. A basal concentration of ROS is indispensable for the manifestation of cellular functions, whereas excessive levels of ROS cause damage to cellular macromolecules such as DNA, lipids and proteins, eventually leading to necrosis and apoptotic cell death. CVD is the main cause of death worldwide with several conditions being affected by oxidative stress. Increased ROS lead to decreased nitric oxide availability and vasoconstriction, promoting arterial hypertension. ROS also negatively influence myocardial calcium handling, causing arrhythmia, and augment cardiac remodeling by inducing hypertrophic signaling and apoptosis. Finally, ROS have also been shown to promote atherosclerotic plaque formation. This review aims at giving an introduction into oxidative stress in CVD, with special focus on endothelial dysfunction, and then examining in detail the role of oxidative stress in the most prevalent of these diseases. Finally, potential nutraceuticals and diets that might be beneficial in diminishing the burden of oxidative stress in CVD are presented.

## 1. Introduction

A variety of cardiovascular diseases have been shown to be associated, at least partially, with an excess production of reactive oxygen species (ROS) [1,2,3,4]. ROS constitute both oxygen free radicals, such as superoxide, hydroxyl radicals, and peroxyl radicals, as well as non-radicals, such as hydrogen peroxide, hypochlorous acid, and ozone [5]. In most cell types, mitochondria are the main drivers of intracellular oxidant production, while other relevant sources are nicotinamide adenine dinucleotide phosphate (NADPH) oxidases (summarized as the NOX family of enzymes). Apart from that, numerous other enzymes such as xanthine oxidase, nitric oxide synthase, cyclooxygenases, cytochrome P450 enzymes, and lipoxygenases as well as other cell organelles, like the peroxisome and endoplasmic reticulum, contribute to intracellular ROS production [6]. Proteins, lipids and DNA are the primary cellular structures affected by ROS and reactive nitrogen species (RNS). The generation of molecular oxygen in the form of ROS is a natural part of aerobic life. Indeed, basal levels of ROS are essential for the manifestation of various cellular functions, such as signal transduction pathways, defense against invading microorganisms, gene expression and the promotion of growth or death [7]. In spite of the crucial relevance of redox reactions, dysregulation of oxidant signaling may cause or accelerate a host of pathological conditions, such as the rate of aging. However, the body is equipped with protective measures against ROS via enzymatic (e.g., superoxide dismutase (SOD), catalase (CAT), peroxiredoxin (Prx) and glutathione peroxidase (GSH-Px)) as well as non-enzymatic compounds (e.g., tocopherol/vitamin E, beta-carotene, ascorbate, glutathione (GSH), and nicotinamide (NAM)) [8]. Newer research tools allow the investigation of redox signaling pathways in adequate chemical detail, and it has become clear that redox processes are as important in biology as phosphorylation-dephosphorylation reactions, or central mechanisms responsible for controlling the genome and epigenome, such as acetylation–deacetylation and methylation–demethylation [9]. However, the analysis of redox systems is challenging due to the substantial subcellular differences in redox potential and the short lifespan of ROS. The discovery of numerous biomarkers of oxidative stress has facilitated the measurement of ROS; however, their clinical use still needs to be validated given the vast diversity in oxidative stress between different diseases. Genomics, epigenomics, and exposomics along with methodologies for redox imaging, redox proteomics, and redox metabolomics, will improve our understanding of health and disease processes within entire biological systems. Furthermore, the emerging big data and artificial intelligence era will provide us with new opportunities for the development of oxidative stress knowledge bases and paves the way for a more personalized redox medicine [9].

In 2016, ≈17.6 million deaths were attributed to cardiovascular diseases (CVD) globally, which amounted to an increase of 14.5% from 2006 [10]. CVD is currently the leading cause of death, and it claims more lives each year than cancer and chronic lung disease combined. Coronary heart disease (CHD) represents the most common CVD [10]. In the coming decades, with an aging population and increased incidence of obesity and diabetes, the burden and medical costs of CVD are anticipated to significantly increase worldwide. Although significant efforts have been made to enlighten pathophysiologic mechanisms governing the initiation and progression of CVD, still much work has to be done [11,12,13]. As such, a better understanding of the biomolecular mechanisms and their clinical consequences is urgently needed to reduce the burden of CVD, and this poses a serious challenge in medicine.

This review will exemplify the role of oxidative stress in CVD, first enlightening the pathomechanisms underlying oxidative stress and CVD with a special focus on a main contributor of disease, namely endothelial dysfunction. Afterwards, it delves into oxidative stress and inflammation in the most prevalent of these diseases and will finally conclude with a discussion about the effects of diet and nutraceuticals on oxidative stress in CVD.

## 2. Endothelial Dysfunction in Cardiovascular Disease

The endothelium is a highly active monolayer that plays important roles in modulating vascular tone, cellular adhesion, thromboresistance, smooth muscle cell proliferation, and vessel wall inflammation. This is achieved by the production and release of several endothelium-derived relaxing factors, including vasodilator prostaglandins, nitric oxide (NO), and endothelium-dependent hyperpolarization factors, as well as endothelium-derived contracting factors. These vasoactive molecules that relax or constrict the vessel play a direct role in the balance of tissue oxygen supply, long-term organ perfusion, remodeling of vascular structures, and metabolic demand by regulation of vessel tone and diameter [14,15]. Endothelial cells dispose of an enzyme to fight vascular disease, namely endothelial nitric oxide synthase (eNOS), which generates the vasoprotective molecule NO. This molecule diffuses to the vascular smooth muscle cells, activates soluble guanylyl cyclase and increases cyclic guanosine monophosphate (cGMP) [16]. NO can also inhibit leukocyte adhesion to the vessel wall which represents an early event in the development of atherosclerosis; therefore, NO may protect against the onset of atherogenesis. Furthermore, NO is also involved in the inhibition of platelet aggregation and adhesion, both of which protect smooth muscle cells from exposure to platelet-derived growth factors. These mechanisms can lead to fibrous plaque formation; therefore, NO also prevents a later step in atherogenesis. NO suppresses key processes in vascular lesion formation and thus probably represents the most important antiatherogenic defense principle in the vasculature [16].

**Figure 1:**
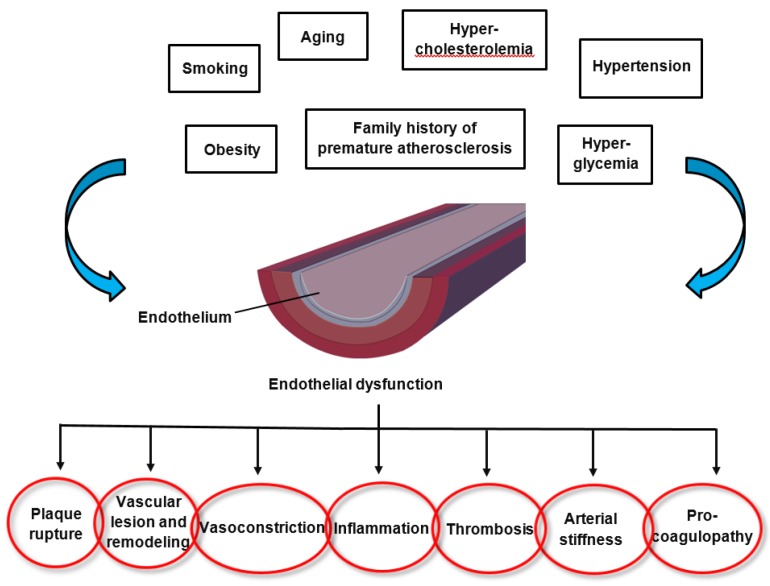
Factors altering endothelial function and the consequences of endothelial dysfunction.

The pathomechanisms of endothelial dysfunction involve a diminished production and/or availability of NO, and a disproportion between the endothelium-derived vasodilators and vasoconstrictors. Several traditional cardiovascular risk factors are associated with alteration in endothelial function such as smoking, sedentary and incorrect lifestyle, aging, hypercholesterolemia, arterial hypertension, hyperglycemia, and a family history of premature atherosclerotic disease (Figure 1) [17,18]. This leads to chronic inflammation, resulting in an increase in vasoconstrictor and prothrombotic products and diminished antithrombotic factors, in addition to abnormal vasoreactivity, all of which elevate the risk of cardiovascular events. Indeed, endothelial dysfunction has also been linked with obesity, elevated C-reactive protein, and chronic systemic infection [18]. Oxidative stress and inflammation are the main drivers of endothelial dysfunction. Several oxidative enzyme systems such as NADPH oxidase, xanthine oxidase, cyclooxygenases, lipoxygenases, myeloperoxidases, cytochrome P450 monooxygenase, uncoupled NOS, and peroxidases lead to the inactivation of NO, which represents a critical mechanism leading to endothelial dysfunction through an elevated level of superoxide anion (O_2_^•−^) [19]. Both NADPH oxidase and eNOS uncoupling (i.e., the generation of ROS through eNOS as part of endothelial activation) act as important sources of O_2_^•−^ that give rise to vascular oxidative stress. Inhibition of NADPH oxidase has been established as a key molecular mechanism leading to reduced arterial oxidative stress and normalization of endothelial dysfunction in mice [20]. Inflammation has been shown in many studies to play a role in endothelial dysfunction that underlies the pathogenesis of CVD, obesity and type 2 diabetes mellitus. Both in rodents and humans, elevated levels of pro-inflammatory cytokines such as tumor necrosis factor-alpha (TNF-α), interleukin-1beta (IL-1 β), interleukin-6 (IL-6), and interferon gamma (IFN-γ) have been observed in age-related endothelial dysfunction, mainly via the activation of the nuclear factor-kappa B (NF-κB) pathway [21,22,23,24,25,26]. NF-κB is an important transcription factor that regulates the gene expression of factors responsible for cell adhesion, proliferation, inflammation, redox status, and tissue specific enzymes. It is expressed in all cell types and plays a major role in the promotion of CVD through the transcription of pro-inflammatory, pro-adhesion and pro-oxidant genes [27]. The NF-κB pathway can be activated by a variety of stimuli including inflammatory cytokines, ROS, lipids and mechanical forces acting on the vascular endothelial wall. Upon activation, transmembrane receptors are stimulated which trigger intracellular signaling pathways, culminating in the activation of a kinase (IκK) mediated phosphorylation and degradation of the inhibitor of NF-κB (IκB). Subsequently, the NF-κB heterodimer (p65/p50 subunits and, perhaps, p65, RelB, c-Rel, p50 and p52) translocates to the nucleus, where it binds to promoters of gene targets. Several other pro-inflammatory molecules have been associated with endothelial dysfunction, such as IL-6, TNF-α, monocyte chemoattractant protein 1 (MCP-1), receptor for advance glycation endproducts (RAGE) and the pro-oxidant enzyme NADPH oxidase, and all predispose the vasculature to a “proatherogenic” phenotype [28,29,30].

The endothelium has an endogenous capacity for repair, which occurs via two mechanisms. Lost and damaged cells can be replaced by locally replicating mature endothelial cells. However, this repair mechanism is insufficient in the presence of risk factors and loss of endothelial integrity would rapidly ensue. Circulating endothelial progenitor cells represent an alternative mechanism for maintenance and repair of the endothelium; they are recruited from the bone marrow. These cells circulate in the peripheral blood and have the ability to differentiate into mature cells with endothelial characteristics [15]. Indeed, factors that have been shown to have a positive impact on endothelial function, such as exercise and statins, have also been shown to have a potent positive effect on the mobilization of endothelial progenitor cells [31,32]. The importance of the balance between exposure to risk factors and the efficiency of endothelial repair has been underscored by the observation that subjects with increased numbers of circulating endothelial progenitor cells have preserved endothelial function, despite exposure to high levels of risk factors [33].

Endothelial therapy can be viewed as a two-step approach. The best treatment for diseases is preventing the disease from occurring in the first place. Therefore, the first approach is disease prevention through increased awareness and control of cardiovascular risk factors by nonpharmacological measures such as lifestyle optimization (e.g., a healthy diet, physical exercise, maintaining a normal body weight). The second approach is targeted (pharmacological) therapy directed at preserving or restoring the function of already impaired endothelial cells in order to defer disease progression, promote disease stabilization, improve overall quality of life, reduce disability and health costs, and ultimately increase survival [34]. The aim of pharmacological treatment is reestablishing endothelial cell homeostasis (e.g., statin therapy to reduce low-density lipoprotein (LDL) cholesterol levels; antidiabetics to reduce blood glucose levels; antihypertensives to normalize blood pressure; heart failure therapy to amend myocardial and vasomotor function). Certain drugs may have some additional, endothelium-protective off-target effects, such as statins by reducing inflammation and some angiotensin antagonists also having metabolic (antidiabetic) effects [35,36]. Researchers are currently exploring new pathogenetic targets to improve vascular dysfunction, including anti-inflammatory agents, therapies based on microRNAs and epigenetic mechanisms. MicroRNAs (miRNAs) have been demonstrated to play a pivotal role during atherosclerotic plaque formation. Indeed, it has been established that both miR-143 and miR-181a are upregulated in human atherosclerotic plaques [37,38]. Hydrogen peroxide (H_2_O_2_) treatment induces an increase in miR-181a levels, while inhibition of miR-181a leads to increased resistance to H_2_O_2_, thus implying that miR-181a is involved in the oxidative stress-induced endothelial cell dysfunction [37]. MiR-133a may represent an additional target for preventing cardiovascular disease. Studies have demonstrated that the inhibition of aberrant miR-133a by lovastatin prevents endothelial dysfunction by targeting GTP cyclohydrolase 1, a critical enzyme for eNOS uncoupling in endothelial dysfunction [39]. Finally, inhibition of miR-92a, an important regulator of endothelial proliferation and angiogenesis after ischemia, leads to reduced endothelial inflammation, decreased plaque size, and a more stable lesion phenotype [40]. In recent years, emerging evidence has arisen that epigenetic pathways might also play an important role in endothelial dysfunction. Resveratrol, a member of the polyphenol group, is produced by several plants in response to injury and protects against pathological processes through the suppression of elevated levels of proinflammatory cytokines in macrophages. Increased TNF-α-induced CD40 expression has been shown to modify the expression levels of specific adhesion molecules, thus boosting the inflammatory response. Resveratrol treatment was able to attenuate the enhanced CD40 expression triggered by TNF-α stimulation. Furthermore, resveratrol suppressed TNF-α-triggered ROS via potentiating the activity of sirtuin 1 (a histone deacetylase involved in suppressing inflammation), thus protecting cells from damage generated by inflammatory factors [41]. 

## 3. Oxidative Stress and Inflammation in Cardiovascular Diseases

The NOX (for **N**ADPH **OX**idase) family NADPH oxidases are transmembrane proteins that transfer a single electron from NADPH onto molecular oxygen, leading to the formation of superoxide. The physiological generation of ROS usually occurs as a byproduct; however, this is not the case with NOX enzymes, as the generation of ROS represents their primary biological function. In fact, the NOX-mediated release of ROS, also known as oxidative burst, promotes the eradication of invading microorganisms in macrophages and neutrophils. The importance of ROS in the host immune response is accentuated by the fact that people with an inherited deficiency in NOX2 develop chronic granulomatous disease (CGD) and are incapable of warding off common infections. The first NADPH oxidase, NOX2, was found in phagocytes. This was followed by the discovery of other members of the NOX family NADPH oxidases, which are not limited to phagocytes, but can de facto be found in virtually every tissue [42]. There is substantial evidence indicating that NOX enzymes play an essential role in the pathophysiology of several CVD [43,44]. 

**Figure 2:**
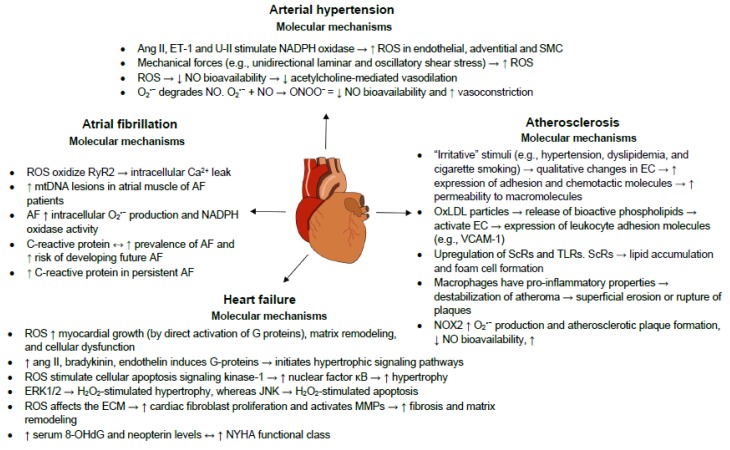
Selected cardiovascular diseases and their underlying oxidative and inflammatory molecular mechanisms. 8-OHdG: 8-hydroxy-2′-deoxyguanosine AF: atrial fibrillation; Ang II: angiotensin II; EC: endothelial cells; ECM: extracellular matrix; ERK 1/2: extracellular signal-regulated kinase 1/2; ET-1: endothelin 1; H_2_O_2:_ hydrogen peroxide; JNK: c-Jun N-terminal kinase; MMP: matrix metalloproteinase; mtDNA: mitochondrial DNA; NADPH: nicotinamide adenine dinucleotide phosphate; NO: nitric oxide; NYHA: New York Heart Association; O_2_^−^: superoxide anion; ONOO^−^: peroxynitrite; OxLDL: oxidatively modified LDL; ROS: reactive oxygen species; RyR2: type 2 ryanodine receptor; ScRs: scavenger receptors; SMC: smooth muscle cells; TLRs: toll-like receptors; U-II: urotensin II; VCAM-1: vascular cell adhesion molecule-1; →: leads to; ↔: associated with. Please refer to the text for more details.

Inflammation is an adaptive reaction to harmful stimuli and certain conditions, such as infection or tissue injury, and comprises the regulated delivery of blood components (plasma and leukocytes) to the site of infection or injury. A contained inflammatory response is generally thought to be beneficial (e.g., granting protection against infection), but can become destructive if dysregulated (e.g., causing septic shock) [45]. We will subsequently discuss the roles of oxidative stress and inflammation in the most prevalent CVD. The underlying oxidative and inflammatory molecular mechanisms governing these CVD are summarized in Figure 2.

### 3.1. Atherosclerosis

Atherosclerosis remains the leading cause of cardiovascular death in developed countries [10]. There is emerging evidence that inflammatory mechanisms play a key role in atheroma formation. Activation of pro-inflammatory signaling pathways, expression of cytokine/chemokine, and increased oxidative stress are some of the mechanisms leading to atherosclerosis [4]. “Offending” stimuli (e.g., dyslipidemia, hypertension and cigarette smoking) lead to qualitative changes in endothelial cells, promoting the expression of adhesion and chemotactic molecules and an increased permeability to macromolecules. This facilitates the entry of LDL particles in the arterial wall and subsequent retention as a result of the binding of apolipoprotein B100 to proteoglycans of the extracellular matrix (ECM) [46]. Oxidatively modified LDL (OxLDL) particles lead to the release of bioactive phospholipids capable of activating endothelial cells. Activated endothelial cells express various types of leukocyte adhesion molecules, such as the vascular cell adhesion molecule-1 (VCAM-1), which mediates the rolling and adhesion of blood leukocytes (monocytes and T cells) [47]. Monocytes then differentiate into macrophages, which represents a crucial step in the pathogenesis of atherosclerosis and is followed by the upregulation of pattern recognition receptors for innate immunity, particularly scavenger receptors (ScRs) and toll-like receptors (TLRs). ScRs incorporate apoptotic cell fragments, bacterial endotoxins, and OxLDL, causing lipid accumulation and foam cell formation [47]. Lectin-like oxLDL receptor-1 (LOX-1) is a multiligand ScR originally recognized as the principal receptor for oxLDL uptake by endothelial cells. However, this receptor is also expressed by monocytes/macrophages, smooth muscle cells, cardiomyocytes, adipocytes, fibroblasts, platelets, and many more. LOX-1, which is expressed in atheroma-derived cells and is widely detected in human and animal atherosclerotic lesions, mediates a range of proatherogenic cellular responses involved in the pathogenesis of atherosclerosis. Some of these responses include endothelial dysfunction, phagocytosis of senescent apoptotic cells, vascular inflammation, foam cell formation, collagen deposition, and adipocyte cholesterol metabolism [48]. TLRs activate macrophages, which release vasoactive molecules such as NO, endothelins, and several eicosanoids, and also mediate the production of ROS [47]. Macrophages play critical roles in atherogenesis through their pro-inflammatory properties that assist in the destabilization of atheroma by degrading ECM and hence promoting erosion or rupture of plaques, which ultimately culminates in occlusive thrombus formation [49]. NOX2, a specific NADPH oxidase isoform, has been identified as a key player in atherogenesis [50]. A study led by Judkins et al. [50] found that NOX2 expression is upregulated in aortic endothelial cells and in macrophages of apolipoprotein E-null (ApoE-/-) mice already before the appearance of atherosclerotic lesions, and these changes coincided with an increased aortic superoxide production. NOX2 deficiency in these mice had a negligible influence on plasma lipid profiles but correlated with a decreased aortic superoxide production, improved NO bioavailability, and pronounced reductions in atherosclerotic plaque formation. Thus, this study provided conclusive evidence that NOX2 plays a pivotal role in increased superoxide production, impaired NO bioavailability, and atherosclerotic plaque formation in ApoE-/- mice.

Interestingly, the atherosclerotic plaque leading to thrombosis does not strictly correlate with the degree of stenosis at angiography, but rather depends upon the cellular features of the plaque and particularly on the density of the overlying fibrous cap. In fact, atherosclerotic plaques susceptible to rupture display increased accumulation of inflammatory cells which leads to the degradation of collagen through the release of collagenolytic enzymes, mainly matrix metalloproteinases (MMPs) and LDL Lp-PLA_2_ (lipoprotein-associated phospholipase A_2_), in addition to reducing its synthesis by promoting smooth muscle cell apoptosis [51].

### 3.2. Heart Failure

Heart failure (HF) is an emerging epidemic disease in the developed world affecting approximately 1% to 2% of the adult population. It is a progressive disease, associated with an annual mortality of approximately 10%. Effective treatment has improved outcomes; however, the prognosis is still poor, with a 5-year mortality rate of 25% to 50% [52]. A variety of experimental and clinical studies have indicated that increased generation of ROS is implicated in the pathogenesis of HF [53,54,55,56]. ROS stimulate myocardial growth, matrix remodeling, and cellular dysfunction by activating various hypertrophy signaling kinases and transcription factors. G proteins (GTP-binding proteins) are heterotrimeric protein complexes consisting of α, β, and γ subunits, and mediate signals from distinct stimuli outside a cell to its interior. Within the myocardium, Angiotensin II (Ang II), bradykinin, endothelin, and α-adrenergic stimulation induce Gα_i_ and Gα_q_ to institute many of the hypertrophic signaling pathways [57]. Although it is well known that G-protein coupled receptor (GPCR) activation can lead to ROS generation, there is data indicating that ROS can directly induce G-protein dissociation and activation [58,59]. In neonatal rat ventricular myocytes, the activation of extracellular signal-regulated kinase 1/2 (ERK1/2), mitogen-activated protein kinase (MAPK) and Akt by H_2_O_2_ required the Gβγ subunits of G_i_ and G_o_, but was independent of ligand binding to GPCR [59]. A consecutive study demonstrated that ROS activate G_i_ and G_o_ by alteration of two cysteine residues (Cys^287^ and Cys^326^). Two steps are required for the activation of G_i_ and G_o_ by ROS. The first step is the oxidation of Cys^287^ of Gα_i2_, leading to subunit dissociation into Gα_i2_ and Gβγ. The second step is the oxidation of Cys^326^, leading to the activation of Gα_i_ [58]. Thus, ROS seem to promote hypertrophic growth signaling by direct activation of G proteins in neonatal rat ventricular myocytes. ROS also stimulate cellular apoptosis signaling kinase-1, a redox-sensitive kinase, which, when overexpressed, leads to NF-κB -induced hypertrophy. Indeed, genetic silencing of apoptosis signaling kinase-1 inhibits hypertrophy generated by Ang II, norepinephrine, and endothelin 1 (ET-1) [60]. H_2_O_2_ causes concentration-dependent effects on adult rat ventricular myocytes (ARVM), leading to hypertrophy at low concentrations and apoptosis at higher concentrations. Activation of four kinase signaling pathways—ERK1/2, c-Jun-N-terminal kinase (JNK), p38 mitogen-activated protein kinase and Akt—is responsible for these effects. H_2_O_2_-induced hypertrophy is mediated by ERK1/2, while apoptosis is mediated by JNK. Finally, ROS-dependent activation of ERK1/2 and Akt exerts anti-apoptotic actions, thus opposing the pro-apoptotic effect of JNK [61]. ROS also affect the ECM, stimulating the proliferation of cardiac fibroblasts and activating MMPs, fundamental effects leading to fibrosis and matrix remodeling [62,63,64]. MMPs are usually secreted in an inactive form and are activated post translationally by ROS [62]. MMPs play a central role in normal tissue remodeling processes, such as cell migration, invasion, proliferation, and apoptosis, and have been demonstrated to be elevated in the failing hearts [65,66]. Indeed, a study has demonstrated significant survival improvements after myocardial infarction (MI) in MMP-2 knockout mice, which could mainly be attributed to a reduced rate of early cardiac rupture and the subsequent development of LV remodeling and failure [67]. Another study examined the effect of oxidative stress in the progression of LV remodeling and failure after MI in mice and whether dimethylthiourea, an ^•^OH scavenger, could debilitate these changes [68]. The activation of MMP-2 was inhibited by dimethylthiourea treatment, which led to a markedly improved LV contractile function and a more moderate increase in LV chamber size and hypertrophy compared with mice not treated with the drug. These findings suggest that increased oxidative stress can act as a stimulus for the activation of myocardial MMP, which is decisively involved in LV remodeling and consequently in the development and progression of HF.

An action potential-induced membrane depolarization and the ensuing Ca^2+^ influx through dihydropyridine-sensitive L-type Ca^2+^ channels leads to the contraction of cardiac myocytes. The proportion of Ca^2+^ released by these channels is relatively small; thus, a subsequent opening of ryanodine receptors (RyRs) ensues, which promotes a substantial Ca^2+^ release from the sarcoplasmic reticulum Ca^2+^ store [69]. ROS have been shown to affect the function of RyRs. Most studies indicate that the oxidation of thiol (SH) groups of the RyR are responsible for the effects of ROS [70]. It has been demonstrated that the open probability of cardiac and skeletal muscle RyRs is increased in the presence of O_2_^−^ and H_2_O_2_/^•^OH and that agents that reduce SH groups are capable of reversing this effect [71,72,73]. There is also data indicating that after the initial stimulation of RyRs, ROS can irreversibly inactivate these receptors [64]. The actions of ROS on these receptors are contingent on the concentration and length of exposure.

In recent years, oxidative stress markers have been increasingly adopted in heart failure patients. One of the biomarkers that have gained increased attention is 8-hydroxy-2′-deoxyguanosine (8-OHdG), which represents one of the prevailing forms of free radical-induced oxidative DNA lesions [74]. A study [75] showed that high serum 8-OHdG levels correlated with more severe New York Heart Association (NYHA) functional class and an increased incidence of cardiac events, although plasma B-type natriuretic peptide level did not differ between high and normal 8-OHdG groups. Interaction of advanced glycation end products (AGEs) and its receptor (RAGE) initiates a signaling cascade that activates the transcription factor NF-κB, inducing oxidative stress and leading to an expanded release of inflammatory cytokines such as TNF-α, thus AGEs and RAGE are recognized as oxidative stress markers [76]. Consequently, in the abovementioned study, they further measured both serum levels of pentosidine, a member of the AGEs, and soluble form of RAGE, and elevated levels of pentosidine and soluble RAGE predicted an increased incidence of cardiac events in heart failure patients. Furthermore, both levels were independent predictors of cardiac events in a multivariate cox proportional hazard analysis. Another biomarker, neopterin, is produced by macrophages mainly after interferon-γ stimulation and is associated with the formation of ROS [77]. In the abovementioned study, a higher serum neopterin concentration in heart failure patients correlated with more severe NYHA functional class and an increased risk of cardiac events. Therefore, the authors concluded that biomarkers of oxidative stress are useful surrogate parameters to identify heart failure patients who are at increased risk.

### 3.3. Arterial Hypertension

The global prevalence of hypertension was estimated to be 1.13 billion in 2015, with an overall prevalence in adults of around 30–45%. The disease becomes more prevalent with advanced age, with >60% of people aged >60 years having arterial hypertension [78]. Cumulated evidence indicates that oxidative stress could be a contributing factor in the pathogenesis of hypertension [79,80].

In the vascular system, no specific NOX isoform exists, but rather an intricate expression of various NOX isoforms in different cells and regions of the vasculature. NOX4 seems to be the predominant isoform in endothelial cells and vascular smooth muscle. The activity of NOX4 is much higher in cerebral arteries compared to systemic arteries. NOX1 and NOX2 have distinct anatomical distributions, with NOX1 mainly being expressed in large conduit vessels, while NOX2 is predominantly found in resistance vessels. The function of NOX-derived ROS in the vasculature is complex and is dependent both on the NOX isoform as well as on the cell type. NOX4 is the most prominently expressed NOX family member in all cells of the cardiovascular system [42]. In the vascular system, ROS are produced in endothelial, adventitial and smooth muscle cells, primarily induced by NADPH oxidase which produces O_2_^•−^ upon being stimulated by Ang II, ET-1 and urotensin II (U-II), among others. Additionally, increased mechanical forces due to elevated blood pressure, such as unidirectional laminar and oscillatory shear stress, can contribute to increased ROS production. ROS can act as intracellular second messengers and contribute to a rise in intracellular Ca^2+^ concentration, leading to vasoconstriction and thereby assisting in the pathogenesis of hypertension [81]. NOX signaling plays a role in endothelium-dependent vasorelaxation which is mainly mediated by NO. Endothelial cells release NO, thereby causing vascular relaxation. NO is rapidly degraded by the oxygen-derived free radical O_2_^−^. Superoxide anions produced by NOXs react with NO to produce peroxynitrite, thereby decreasing the bioavailability of NO and inducing vasoconstriction. Therefore, hypertension is associated with a reduced availability of NO and increased oxidative stress [82]. Ang-II-induced hypertension involves the activation of redox-dependent signaling cascades and NADPH oxidase-induced generation of ROS [83]. Ang II, the major bioactive peptide of the Renin-Angiotensin System (RAS), plays an important role in many vascular processes such as vasoconstriction, hypertrophy, fibrosis, inflammation, and aging. Vascular NOXs are all regulated by Ang II and NOX-derived ROS signaling mainly mediate the Ang II effects [84]. Ang II activates the Ang 1 receptor, which increases the production of O_2_^−^ via membrane-bound NADPH-driven oxidases [85]. Indeed, chronic Ang II infusion in rats has been shown to increase the activity of vascular NADPH oxidase, thereby inducing hypertension [86]. Some common antihypertensive medication, such as Ang 1 receptor blockers and angiotensin-converting enzyme (ACE) inhibitors, have been shown to exert their antihypertensive action in part by inhibiting NADPH oxidase and decreasing ROS production [82]. It is well established that excessive oxidative stress and a diminished capacity for scavenging free radicals contributes to hypertension. Furthermore, in hypertensive subjects, systolic and diastolic blood pressure have been shown to positively correlate with oxidative stress markers and negatively correlate with plasma antioxidant capacity [87].

### 3.4. Atrial Fibrillation

Atrial fibrillation (AF) represents the most common arrhythmia in clinical practice. The risk for atrial fibrillation increases with aging, occurring in fewer than 1% of persons aged 60 to 65 years but in 8% to 10% of those older than 80 years [88]. Both human and animal data have established the role of oxidative stress in the pathogenesis of AF [89,90,91], which is substantiated by the fact that antioxidant drugs are capable of positively influence the development of AF [89,90,92]. The type 2 ryanodine receptor (RyR2) constitutes the primary intracellular Ca^2+^ release channel in atrial myocytes, and dysfunction of this channel caused by oxidative stress alters intracellular Ca^2+^ homeostasis, a phenomenon implicated in the pathogenesis of AF [93]. Transgenic mice disposing of a constitutively leaky RyR2 channel, which displays a RyR2 oxidation in ventricular myocytes, have been studied to examine the role of intracellular Ca^2+^ leak via this channel in the development of AF [94]. The atrial RyR2 has been shown to represent a target of oxidative stress and as such is involved in the pathogenesis of AF. In atrial myocytes, RyR2 are being oxidized by mitochondrial-derived ROS, which causes an increased leak of intracellular Ca^2+^. Of note, it has been shown that mitigating the production of ROS reduces atrial diastolic Ca^2+^ leak, thus impeding the development of AF [94].

Human mitochondrial DNA (mtDNA) is highly susceptible to oxidative damage and mutation due to poor proofreading and inefficient DNA repair during replication. Aging human tissues exposed to increased oxidative stress have been reported to frequently exhibit a common 4977 base pair deletion in their mtDNA [95]. In a study conducted by Lin et al. [96], the authors hypothesized that enhanced oxidative injury and mutations in the mtDNA may be involved in the development of AF. By means of the polymerase chain reaction (PCR) technique, they found that the occurrence of mtDNA with 4977 bp deletion was 3.75 times higher in the atrial muscle of AF patients compared with patients without AF. Furthermore, they examined the oxidative damage to mtDNA by measuring the level of mtDNA lesions in atrial muscle, which was markedly increased in AF patients. Thus, they contributed to the existing knowledge that oxidative injury in the cardiac muscle is increased in AF patients.

Dudley et al. [97] provided further evidence that oxidative stress is implicated in the pathogenesis of AF. For one week, they induced AF in pigs by rapid atrial pacing and measured afterwards the O_2_^•−^ production from acutely isolated heart tissue using two independent techniques, electron spin resonance and superoxide dismutase–inhibitable cytochrome C reduction assays. In the experimental animals, to maintain AF, they programmed the device to a rate of 600 beats per minute (bpm), while a separate single-chamber pacemaker was used with the ventricular lead programmed to a rate of 100 bpm. In the control group, a single DDD pacemaker was used with both atrial and ventricular leads programmed to 100 bpm. In this way, the paced ventricular rate was 100 bpm in both groups, and they differed merely in the paced atrial rhythm. In the experimental animals, 1 week of AF increased the intracellular O_2_^•−^ production by 2.7- and 3.0-fold in the left atrium (LA) and left atrial appendage (LAA), respectively, compared with the control group. No changes in O_2_^•−^ production in the right atrium or right atrial appendage were observed. Similarly, the extracellular O_2_^•−^ production in the LAA increased 3-fold in the experimental group as opposed to the control group. Furthermore, the NADPH oxidase activity was measured, which was substantially increased both in the LA and LAA compared with the control group. In summary, the authors demonstrated that, in a pacing model of AF, increased oxidative stress is confined to the LA and LAA and that at least some of the O_2_^•−^ produced by the atria can be attributed to the NADPH oxidase.

Inflammation also undoubtedly contributes to the development of AF [98,99]. However, it is well established that inflammation and oxidative stress are closely intertwined and that one amplifies the other. C-reactive protein, a marker of inflammation, has been shown to correlate with both the presence of AF and the risk of developing future AF. Furthermore, patients with persistent AF have a higher C-reactive protein level than those with paroxysmal AF. Several drugs such as statins, glucocorticoids, ACE inhibitors, and angiotensin receptor blockers have been shown to reduce the recurrence of AF, which can partially be explained by their anti-inflammatory activity [99].

## 4. Effects of Diet and Nutraceuticals on Oxidative Stress in Cardiovascular Diseases

Based on the prevalence of CVD and the role of ROS in many pathologies, as specified above for the cardiovascular system, there has long been interest in the application of naturally occurring antioxidants and the development of chemical antioxidative agents to ease or prevent CVD. The subsequent chapter outlines available evidence regarding the effect of diet and nutraceuticals, i.e., the beneficial effects that substances contained in foods have on human health [100], on the prevention and therapy of oxidative stress in distinct CVD (Table 1).

### 4.1. Polyphenols

Polyphenols, a class of natural, synthetic and semi-synthetic substances, are composed of large multiples of phenol units and have been shown to reduce the risk of cardiovascular mortality, myocardial infarction and stroke [135,136].

#### 4.1.1. Olive oil

Extra virgin olive oil (EVOO) and cocoa are rich in polyphenols [101,137]. A study [101] examined the antioxidant effects of EVOO in healthy subjects by comparing meals containing EVOO with meals containing corn oil. When a meal containing corn oil was given, platelet ROS generation, platelet and serum sNOX2-dp release, and 8-iso-PGF2α-III formation all increased significantly compared to baseline, while vitamin E levels decreased. Furthermore, sVCAM1 and E-selectin levels, markers of endothelial dysfunction, also increased significantly. Conversely, in subjects eating a meal containing EVOO, a non-significant increase of platelet ROS generation, platelet and serum sNOX2-dp release, 8-iso-PGF2α-III formation, sVCAM1 and E-selectin levels and a non-significant decrease in vitamin E levels were found. Additionally, the authors conducted an in vitro study using platelets as a cellular source of ROS-derived NOX2 activation. They showed that platelets incubated with EVOO markedly reduced oxidative stress as confirmed by decreased cell ROS and 8-iso-PGF2-III formation and NOX2 regulation, substantiating the notion that EVOO specifically affects ROS-derived NOX2. In summary, subjects given EVOO demonstrated markedly lower levels of markers of oxidative stress and endothelial dysfunction as compared to subjects given corn oil. Given that olive oil has a higher polyphenol content than corn oil, the different impact of both oils on post-prandial oxidative stress supports the assumption that polyphenol may partially contribute to this effect.

#### 4.1.2. Cocoa

Dark chocolate contains a greater amount of cocoa compared with milk chocolate. A study sought to examine the antioxidant properties of cocoa on platelet-derived oxidative stress and platelet activation [102]. Twenty healthy subjects (HS) and twenty smokers were randomly assigned to receive dark (cocoa >85%) or milk chocolate (cocoa <35%). At baseline, smokers displayed an elevated platelet production of ROS, sNOX2-dp and 8-iso-PGF2a and reduced platelet NOx compared to HS, while the production of platelet TxB2 was marginally increased in smokers. Following the consumption of dark chocolate, a change in platelet oxidative stress could only be observed in smokers. The latter group showed, compared with baseline, reduced platelet ROS and NOX2 activation, and a lower platelet production of 8-iso-PGF2a, while platelet NOx levels increased. No change in platelet TxB2 was observed. Milk chocolate had no effect on platelet oxidative stress, eicosanoid production and platelet function in either of the two groups. This study demonstrated for the first time that NOX2 in platelets is upregulated in smokers, underscoring the notion that smoking leads to increased oxidative stress. Furthermore, they showed that a phenol-rich nutrient, namely dark chocolate, but not milk chocolate, reduces oxidative stress by down-regulating platelet NOX2 and diminishing platelet activation via lowering 8-iso-PGF2a formation. However, these effects were only observed in smokers, implicating that an elevated baseline ROS level is necessary to reduce oxidative stress by compounds disposing of antioxidant property.

#### 4.1.3. Nuts

Nuts are unparalleled among plant foods due to their distinct nutrient composition, which comprises complex carbohydrates, unsaturated fat, protein, fiber vitamins, non-sodium minerals, phytosterols, and other bioactive compounds such as polyphenols [138]. Regular consumption of nuts has been associated with a decreased risk of CVD, overweight and obesity, DM, and cancer [139]. In vitro studies found that nut extracts inhibit LDL oxidation and lipid peroxidation in LDL particles [103,104,105]. One study observed a marked decrease in the formation of thiobarbituric acid reactive substances (TBARS) in human plasma after 4h of incubation in the presence of walnut phenolic extracts [105]. Finally, two in vitro studies showed a protective effect of nuts against strand breaks in the DNA [103,106]. In animal studies, the evidence is not so robust regarding the antioxidant properties of nuts. Two studies measuring lipid peroxidation and antioxidant enzymatic activities did not show a beneficial effect of nut consumption [140,141]. However, one of these studies reported an inverse, dose-dependent association between dietary walnut consumption and aortic ET-1 levels [141]. In contrast, other animal studies reported that nuts increase the activities of serum paraoxonase-1 and arylesterase [107], two antioxidant enzymes, and reduce lipid peroxidation in the plasma, liver, and aorta [108]. The results from human studies investigating the antioxidant capacity of nuts are mixed. One study reported a marked increase in the plasma antioxidant potential in individuals consuming pistachio [109], while other studies did not observe an alteration in the plasma antioxidant capacity subsequent to nut consumption [142,143]. In human studies, nuts have been shown to impact lipid peroxidation, as measured by the mean malondialdehyde (MDA) concentration [109,110,111], while this effect could not be observed when the oxidized LDL in serum was measured [144,145,146]. However, nuts have demonstrated the capacity to enhance endothelium-dependent vasodilation and preserve the protective phenotype of endothelial cells [145,146]. Finally, two studies assessed the impact of nuts on oxidative DNA damage [111,112]. Almond consumption in smokers significantly reduced the amount of DNA strand breaks in lymphocytes and 8-hydroxydeoxyguanosine concentrations in the urine. In conclusion, the positive effects of several types of nuts on oxidation status are inconsistent among in vitro, animal and human studies. Nevertheless, none of the published studies reported any deleterious effects in association with the consumption of nuts.

#### 4.1.4. Tea 

Tea is a widely consumed beverage worldwide [113]. Tea polyphenols, also called catechins, consist of about 30 kinds of phenolic compounds, of which epigallocatechin-3-gallate (EGCG) makes up about 50%–70% of all the catechins. A variety of in vitro and in vivo studies have demonstrated that tea polyphenols possess antioxidant capacities [113,114,115]. Recently, the antioxidant capacity and the phytochemical composition of 30 Chinese teas were evaluated using the ferric-reducing antioxidant power (FRAP) assay and the Trolox equivalent antioxidant capacity (TEAC) assay, two commonly used assays for evaluating the antioxidant capacity [116]. The total FRAP values, which measure the ferric-reducing antioxidant power, differed significantly between the various teas with up to a 9-fold difference. Green teas exerted the highest FRAP values, followed by yellow, dark, oolong, black and white teas. Additionally, water-soluble teas showed the highest reducing capacity, followed by bound-soluble and fat-soluble. Similar to the FRAP values, the TEAC values, representing the free radical-scavenging capacity, also differed markedly between the various teas. Green teas exerted the highest free radical-scavenging capacity, followed by yellow, oolong, dark, black and white teas. The total phenolic content differed 7-fold, with green tea exhibiting the highest content of phenolic compounds, followed by yellow, oolong, dark, black and white teas. Both FRAP and TEAC values significantly correlated with the total phenolic content, which implies that polyphenols are the main contributors to the antioxidant activities of tea [116]. The green tea catechin EGCG has been shown to mitigate the progression of heart failure in mice deficient of the manganese SOD antioxidant enzyme [117]. Mice receiving EGCG showed a better survival and reduced levels of myocardial oxidative stress and free fatty acids compared with mice consuming water. EGCG furthermore prevented the expression of several enzymes and receptors known to promote inflammation. Finally, EGCG prevented the shortening of the telomere length [117]. EGCG has also been shown to prevent heart failure by up-regulating the cardiac sarcoplasmic reticulum Ca-ATPase (SERCA2a), a major regulator of cardiac function. EGCG increased the expression of SECA2a by modifying histone acetylation [118]. The antioxidant properties of tea polyphenols have also been verified in human studies. Catechins have demonstrated the capacity to increase the activity of antioxidant enzymes such as catalase and SOD, scavenge free radicals, and reduce lipid peroxidation. They also contribute to the induction of apoptosis of vascular smooth muscle cells by enhancing the expression of p53, p21, and NF-κB, thus reducing the risk of developing atherosclerosis [119]. A meta-analysis including 13 randomized controlled trials assessed the effect of green tea consumption on blood pressure [120]. Overall, they found that green tea significantly reduces both systolic and diastolic blood pressure, although the net effect was a reduction in both values of about 2 mmHg. Finally, green tea consumption has also been associated with a reduction in mortality in a large, prospective cohort-study with a follow-up of 11 years [121]. Consumption of green tea was inversely associated with all-cause and cardiovascular mortality, while consuming black or oolong tea did not result in a reduced mortality. The reduction in cardiovascular mortality was more pronounced than that of all-cause mortality, with increased tea consumption frequency leading to a greater risk reduction. However, green tea did not reduce cancer mortality.

### 4.2. Flavonoids

Flavonoids are bioactive, polyphenolic compounds which can be found in fruits, vegetables and other vascular plants and cannot be synthesized by humans [147]. Flavonoids have been shown to possess many antioxidant properties, and some of the mechanisms include: direct scavenging of ROS, activation of antioxidant enzymes, metal chelating activity, mitigation of α-tocopherol radicals, inhibition of oxidases, reduction of oxidative stress caused by NO, increase in uric acid levels, and improved antioxidant properties of low molecular antioxidants [148]. An in-depth discussion of these mechanisms is beyond the scope of this review. Flavonoids have been shown to exert antihypertensive properties by targeting different aspects of the NO pathway. Different flavonoids show different modes of action, e.g., luteolin enhances the production of acetylcholine-induced NO, while buddleoside suppresses acetylcholinesterase activity [122]. A systematic review of randomized controlled trials [123] analyzed the impact of flavonoids on vascular function, specifically flow-mediated dilation (FMD) and blood pressure. In pooled analysis of all flavonoid interventions, FMD improved both acutely and chronically and this effect was more pronounced for several flavonoid subclass constituents. Specifically, epicatechin, catechin and procyanidins had a greater acute FMD response compared with analyses that grouped subclasses together. Similar results have been reported for the chronic FMD response, even though the response was more pronounced in the acute phase. In regard to blood pressure, flavonoids have been shown to significantly reduce both systolic and diastolic blood pressure. Again, the response was greatest for specific flavonoid subclasses, such as epicatechin, quercetin and procyanidins. Furthermore, a linear dose response has not been observed for either acute or chronic FMD and blood pressure. However, non-linear dose–response associations have been reported for total flavonoids, procyanidins and epicatechin. Anthocyanins, a type of flavonoid, have been shown to exert potent antioxidant activities and positively impact the function of the vascular endothelium [149]. Their effect on vascular function has been analyzed in a systematic review and meta-analysis of randomized controlled trials [124]. Anthocyanin supplementation improved both acute and chronic FMD but did not improve reactive hyperemia index. Arterial stiffness, as measured by the pulse wave velocity, significantly improved in acute anthocyanin supplementation compared with placebo, while the chronic effects on arterial stiffness were not consistent, with fewer than 50% of the studies reporting improvements in pulse wave velocity. In summary, anthocyanins improve vascular health, especially vascular reactivity as measured by FMD, but may have no significant benefit on arterial stiffness. Quercetin, a dietary antioxidant flavonoid, has been investigated in an animal study [150] regarding its protective and therapeutic effects on lipopolysaccharide (LPS)-induced oxidative stress and vascular dysfunction. Mice treated with LPS had a significantly increased iNOS expression, increased urinary nitrate/nitrite levels, and a reduced expression of eNOS. Quercetin enhanced the eNOS expression in the aortic tissues and reduced the level of nitrate/nitrite, whereas the expression of iNOS was virtually abolished. LPS-injected mice showed a significantly increased aortic O_2_^•−^ production, which could be suppressed with quercetin treatment. Furthermore, quercetin markedly reduced lipid peroxidation and protein oxidation. Finally, mice challenged with LPS showed a dramatically reduced redox ratio of GSH/glutathione disulfide (GSSG), and quercetin treatment regained the GSH redox ratio. There is also human data on the effects of flavonoids. A prospective study assessed the association of flavonoid intake and cardiovascular mortality [125]. Study participants were questioned about their dietary habits and the frequency of consumed foods was noted. Then the daily flavonoid nutrient intake values were estimated. Compared with the lowest quintile, study participants with total flavonoid intakes in the highest quintile had an 18% reduced risk of cardiovascular mortality, even after adjustment for several CV risk factors.

### 4.3. The Mediterranean Diet

The traditional Mediterranean diet (MD) is defined by a high intake of vegetables, fruits, legumes, nuts, cereals, and olive oil but a low consumption of saturated lipids, a moderate intake of fish and dairy products, a reduced consumption of meat and poultry, and a moderate intake of ethanol, mainly wine [151]. An increased adherence to the MD has been associated with a decreased risk of developing CVD, which is thought to stem in part from the antioxidant capacity of the diet to reduce oxidative stress and inflammation [126,127]. Specifically, the MD has been shown to have a positive impact on the lipid metabolism by reducing LDL, apolipoprotein B and A-1, and triglyceride levels, and impeding cholesterol production. Markers of oxidative stress, such as urinary-8-isoprostane and F2-isoprostane, were reduced in individuals consuming a MD, although this was not consistent throughout all studies. Several inflammatory cytokines, such as TNF-α, CRP, IL-1 and IL-6 levels, decreased, while adiponectin levels increased. Some, but not all studies, reported improved insulin sensitivity in individuals consuming a MD [126]. Furthermore, the MD can improve endothelial function as measured by specific markers (FMD and intercellular adhesion molecule 1 (ICAM-1)) [127]. A study of monozygotic and dizygotic middle-aged twins raised in the same family examined the association between adherence to the Mediterranean diet and the plasma GSH/GSSG level [128]. Animal studies have established that the plasma ratio of GSH to GSSG (GSH/GSSG) declines in response to tissue oxidative stress [152]. To calculate the GSH/GSSG ratio, fasting plasma GSH and GSSG concentrations were measured, with a reduced ratio indicating higher oxidative stress. Adherence to the Mediterranean diet was measured using a Mediterranean diet score as previously reported [153]. They found an inverse association between adherence to the Mediterranean diet and oxidative stress, mainly due to lower GSSG concentrations, even after adjusting for various CV risk factors. The association persisted when either monozygotic or dizygotic twins were assessed independent from each other, which indicates that the effects observed in the study are independent of shared familial and genetic factors. Therefore, this study indicates that the mechanism through which the Mediterranean diet confers cardioprotection is through the reduction of oxidative stress.

### 4.4. Polyunsaturated Fatty Acids

Fatty acid (FA) molecules vary in their carbon chain length, possess a methyl terminus and a carboxylic acid head group and are categorized based on the degree of saturation of their carbon chains. Saturated FAs vary from polyunsaturated FAs (PUFAs) in that they possess the maximal number of hydrogen atoms, while the latter have two or more double bonds. Moreover, PUFAs can be classified as either n-6 or n-3, depending on the location of the first double bond relative to the methyl terminus. The last carbon in the FA chain has also been designated the omega carbon, hence the denomination omega-3 or omega-6 PUFAs [154].

Since a correlation between the high intake of *n*-3 PUFAs and the reduced incidence of CVD in Greenland Eskimos was observed, numerous studies have reported beneficial effects of n-3 PUFAs, such as anti-atherogenic, anti-thrombotic and blood pressure-lowering effects. Eicosapentaenoic acid (EPA) and docosahexaenoic acid (DHA), two *n*-3 PUFAs, have not only been shown to reduce plasma triglyceride levels, but also to reduce inflammation and improve endothelial function, thus counteracting the development of atherosclerosis [129,130,131]. For example, n-3 PUFA-derived lipid mediators, namely resolvins, protectins and maresins, have been ascribed anti-inflammatory properties [132]. A specific *n*-3 PUFA receptor, the G-protein-coupled receptor 120, has been described to mediate anti-inflammatory and insulin-sensitizing effects in monocytes/macrophage and adipocytes [133]. A recent study [134] examined the effects of EPA and DHA on DNA damage in human aortic endothelial cells regarding their antiatherogenic mechanisms. A major finding of this study was that EPA and DHA reduced ROS-induced DNA damage in endothelial cells. Double-strand breaks represent the most severe type of DNA damage, and both EPA and DHA reduced H_2_O_2_-induced γ-H2AX foci formation, the most notable marker of double-strand breaks. Key kinases that induce DNA damage, such as H_2_O_2_-generated activation of ATM and DNA-PKcs, were also diminished by n-3 PUFA treatment. Additionally, the intracellular ROS level decreased with EPA or DHA treatment. These data indicate that the genome-protective effects of EPA and DHA observed in the study lie in the reduction of inducers of DNA damage (ROS), rather than assisting the DNA repair system.

### 4.5. Other Food Components

No single antioxidant or beneficial molecule can account for all the salutary effects observed when such diets are being employed. The synergy of multiple molecules, such as polyphenols, flavonoids, minerals (e.g., selenium), carotenoids, and/or melatonin likely represents the underlying mechanism leading to improved cardiovascular health. However, it is difficult to ascertain with certainty which specific food components are responsible for the observed beneficial effects. Therefore, a diet composed of various foods containing different antioxidants, such as fruits and vegetables, herbs, spices, nuts, dark chocolate, olive oil, and fish is recommended over the consumption of supplements consisting of a single antioxidant. Furthermore, it is important to emphasize that natural sources of antioxidants should be preferred over synthetic supplements.

## 5. Benefits and Harms of Antioxidants

Although some diets and nutraceuticals have shown antioxidant properties and even a reduction in cardiovascular morbidity and mortality, several trials investigating various antioxidants have shown no benefit or even harm. A meta-analysis examining the effect of antioxidant supplementation on mortality and health in 66 randomized trials found a positive outcome in 24, a null outcome in 39, and a negative outcome in 3 trials [155]. However, they found that the positive outcome studies included mostly individuals at risk of malnutrition. The outcome of studies examining antioxidant supplements will substantially depend on the nature of the cohort, including the age of the participants, their health status, economic status, toxicity of drug treatments etc. The authors found a benefit of antioxidant supplements mainly in those populations who are at risk for micronutrient deficiencies. Vitamins C and E, selenium, beta-carotene, as well as other nutrients, such as zinc, contribute to the antioxidant defense system. Therefore, individuals with inadequate intake of these vitamins and nutrients may benefit most from antioxidant supplementation. In contrast, individuals with an adequate nutrient status may have reached a threshold, and further antioxidant supplementation is unlikely to confer additional benefit. This threshold varies between each individual and is contingent on an individual’s specific requirements with age, sex, health status, and nutrigenomic factors such as polymorphisms likely altering these requirements [155].

It has been suggested that the indiscriminate use of antioxidants may even be harmful [156]. As already noted in the introduction, free radical production occurs continuously in all cells as part of aerobic life and indeed, basal levels of ROS are imperative for certain cellular functions [7]. Decreasing the amount of free radicals in our organism may impede some essential defense mechanisms such as apoptosis, phagocytosis, and detoxification [157,158]. A fine balance between the magnitude of oxidative stress and antioxidants in our cells is of paramount importance for normal function in the cells and interfering with this balance may lead to untoward effects. The amount of antioxidants that may lend protection varies among individuals and likely depends upon their current oxidative status, as people subjected to increased oxidative stress or those with innate or acquired high baseline levels of ROS may have elevated antioxidant requirements. Conversely, for people with low innate levels of ROS, antioxidant supplementation may even be harmful [157,158]. Additionally, it has been proposed that endogenous antioxidant defenses may be far more important to humans than antioxidants gained from supplements or diet. Therefore, agents that increase endogenous antioxidant defenses and other protective systems may bring about a better protective effect on tissue damage caused by oxidative stress than the consumption of large amounts of vitamins [159].

## 6. Conclusions and Future Directions

Increased oxidative stress has been viewed as one of the potential common etiologies in various CVD. There is substantial evidence for the involvement of free radicals (ROS/RNS) in the pathophysiology of these health disorders. Already, much is known about the molecular mechanisms of oxidative stress that lead to CVD. These diseases are very complex in their pathogenesis, and no single mechanism explains the pathophysiology of these conditions. Therefore, oxidative stress and inflammation need to be viewed as contributing factors and not as the primary pathophysiologic mechanisms. Thus, not surprisingly, many clinical trials investigating antioxidants have been negative. The benefit of antioxidant agents varies based on the oxidative status of each individual, with people having increased levels of oxidative stress benefitting more than people with already low amounts of ROS, as ROS also play a crucial physiologic role in cell homeostasis. Another important factor to take into consideration is the nutritional status of each individual. A balanced diet in high-income countries provides more than sufficient amounts of vitamins, thus further vitamin supplementation is unlikely to confer any benefit and may in fact induce harm. Of notice, however, recent trials have shown a reduced mortality with antioxidant therapies (e.g., green tea, flavonoids, the Mediterranean diet). Such trials, taking into consideration strengths and limitations, do show that oxidative stress plays a pivotal role in CVD and that reduction of oxidative stress reduces cardiovascular and all-cause mortality. These results should encourage scientists to continue to conduct research in the field of oxidative stress and antioxidants. Negative trials about antioxidant agents should not discourage scientists, because in fact, oxidative stress still represents a therapeutic target in CVD.

## Figures and Tables

**Table 1:** Diets and nutraceuticals investigated regarding their potential therapeutic antioxidant effects.

Study Reference	Diet/Nutraceutical(s)	Studied Population	Main Therapeutic Antioxidant Effects
[101]	Extra virgin olive oil	Human and in vitro studies	Stable platelet ROS generation, platelet and serum sNOX2-dp release, 8-iso-PGF2α-III formation, sVCAM1 and E-selectin levels and a NS ↓ in Vit E levels In vitro ↓ cell ROS and 8-iso-PGF2-III formation and NOX2 regulation
[102]	Dark chocolate (cocoa)	Human study	↓ platelet ROS and NOX2 activation, ↓ platelet production of 8-iso-PGF2a, ↑ platelet NOx levels after dark chocolate consumption in smokers
[103,104,105,106,107,108,109,110,111,112]	Nuts	In vitro, animal and human studies	↓ lipid peroxidation, ↓ LDL oxidation, ↓ formation of TBARS, ↓ ROS-induced DNA strand scissions ↑ increase serum paraoxonase-1 and arylesterase activities ↓ DNA strand breaks in lymphocytes and 8-hydroxydeoxyguanosine urine concentrations
[113,114,115,116,117,118,119,120,121]	Tea polyphenols	In vitro, animal and human studies	Scavenging of superoxide and other ROS, inhibition of lipid peroxidation ↑ plasma antioxidant capacity ↓ blood pressure, heart failure and atherosclerosis risk
[122,123,124,125]	Flavonoids	Animal and human studies	↑ acetylcholine-induced nitric oxide production, ↓ acetylcholinesterase activity ↑ eNOS expression and GSH/GSSG ratio ↑ flow-mediated dilation, ↓ blood pressure ↓ NOx, iNOS expression and O_2_^•−^ production ↓ lipid peroxidation and protein oxidation
[126,127,128]	The Mediterranean diet	Human studies	↑ adherence to the MD associated with ↓ oxidative stress and inflammation and ↑ endothelial function and insulin sensitivity ↑ diet score associated with ↑ GSH/GSSG ratio
[129,130,131,132,133,134]	PUFAs	In vitro, animal and human studies	↑ endothelial progenitor cells, ↓ endothelial microparticles inhibition of TLR and TNF-α pro-inflammatory signaling pathways, ↑ insulin sensitivity ↓ ROS-induced DNA damage, ↓ double-strand breaks, ↓ activation of kinases that initiate DNA damage response

8-OHdG: 8-hydroxy-2′-deoxyguanosine; 8-iso-PGF2α-III: 8-iso-prostaglandin F2α; eNOS: endothelial nitric oxide synthase; GSH: glutathione; GSSG: glutathione disulfide; iNOS: inducible nitric oxide synthase; LDL: low-density lipoprotein; MD: Mediterranean diet; NOx: the nitric oxide metabolites nitrite and nitrate; NOX2: NADPH oxidase 2; NS: not significant; PUFAs: polyunsaturated fatty acids; ROS: reactive oxygen species; sVCAM1: vascular cell adhesion molecule 1; sNOX2-dp: soluble NOX isoform 2; TBARS: thiobarbituric acid reactive substances; TLR: toll-like receptor; TNF: tumor necrosis factor; Vit.: vitamin.

## Abbreviations

8-OHdG	8-hydroxy-2‘-deoxyguanosine
O_2_^−^	Superoxide anion
AF	Atrial fibrillation
AGEs	Advanced glycation end products
Ang II	Angiotensin II
CAT	Catalase
cGMP	Cyclic guanosine monophosphate
CHD	Coronary heart disease
CVD	Cardiovascular disease
ECM	Extracellular matrix
ET-1	Endothelin-1
eNOS	Endothelial nitric oxide synthase
ERK 1/2	Extracellular signal-regulated kinase 1/2
EVOO	Extra virgin olive oil
GPCR	G-protein coupled receptor
GPX4	Glutathione peroxidase 4
GSH	Glutathione
GSH-Px	Glutathione peroxidase
GSSG	Glutathione disulfide
H_2_O_2_	Hydrogen peroxide
IL	Interleukin
iNOS	Inducible nitric oxide synthase
INF-γ	Interferon gamma
JNK	c-Jun N-terminal kinases
LOX-1	Lectin-like oxLDL receptor-1
MAPK	Mitogen activated protein kinase
MCP-1	Monocyte chemoattractant protein 1
MD	Mediterranean diet
MDA	Malondialdehyde
miRNA	Micro ribonucleic acid
MMP	Matrix metalloproteinase
MSG	Monosodium-L-glutamate
mtDNA	Mitochondrial deoxyribonucleic acid
NAC	N-Acetyl-L-Cysteine
NADPH	Nicotinamide adenine dinucleotide phosphate
NF-κB	Nuclear factor kappa-light-chain-enhancer of activated B cells
NO	Nitric oxide
NOX2	Nicotinamide adenine dinucleotide phosphate oxidase isoform 2
OxLDL	Oxidatively modified low-density lipoprotein
Prx	Peroxiredoxin
PUFAs	Polyunsaturated fatty acids
RAGE	Receptor for advance glycation endproducts
RAS	Renin-Angiotensin System
RCS	Reactive carbonyl species
ROS	Reactive oxygen species
RNS	Reactive nitrogen species
RyR	Ryanodine receptor
ScR	Scavenger receptor
SMC	Smooth muscle cell
SOD	Superoxide dismutase
TLR	Toll-like receptor
TNF-α	Tumor necrosis factor alpha
U-II	Urotensin-II
VCAM	Vascular cell adhesion molecule-1
